# Alienation from medical care policy, medical care avoidance, and the role of sex and risk perception

**DOI:** 10.1186/s12888-023-05104-0

**Published:** 2023-08-15

**Authors:** Chun Xia, Jia Xu, Xiuzhen Ding

**Affiliations:** 1 Present Address: School of Educational Science, Anhui Normal University, Jiuhua-Nan-Road 189, Wuhu, Anhui Province 241000 China; 2 School of Marxism, Anhui Normal University, Jiuhua-Nan-Road 189, Wuhu, Anhui Province 241000 China; 3 Present Address: School of History, Anhui Normal University, Jiuhua-Nan-Road 189, Wuhu, Anhui Province 241000 China

**Keywords:** SPA-M, Medical care avoidance, Medical financing risk perception, Sex, Moderated mediation model

## Abstract

**Background:**

Medical care avoidance affects individuals’ health status. Previous studies on medical care avoidance have mainly focused on medical costs and people’s satisfaction with medical services. This study investigates whether an individual’s sense of policy alienation toward medical care policy (SPA-M) affects behavioral intention of medical care avoidance, and to what extent an intermediary variable—medical financial risk perception–mediates the relationship between SPA-M and medical care avoidance.

**Methods:**

A cross-sectional survey was conducted involving 434 people aged 35–59 years from Wuhu, a city in China’s Anhui province. A moderated mediation model was constructed to investigate the research question and sex (biological: male and female) was used as a moderating variable between SPA-M and medical financial risk perception.

**Results:**

We found that SPA-M significantly impacted medical care avoidance, and that medical financial risk perception played a complete mediating role in this relationship, while sex moderated the relationship between SPA-M and medical financial risk perception.

**Conclusion:**

This study contributes to the literature by enhancing our understanding of the factors that influence behavioral intention regarding medical care avoidance, deepening our understanding of the role of SPA-M in medical care policy, and expanding the role of sex differences in the analysis of the relationship between SPA-M, medical financial risk perception, and medical care avoidance, offering implications for public and community health.

## Background

Medical care avoidance can result in delayed screening, missed opportunities for optimal recovery, increased risk of developing a serious disease, increased family care burden, increased medical care costs [1–4], and eventually, a decrease in the healthcare condition, quality of life, and life satisfaction of the population, particularly those with chronic diseases [3, 4]. In 2021, 15.3% of Chinese chronic disease patients (including heart and chronic respiratory diseases, cancer, and diabetes), aged 30–70 years, suffered premature mortality due to medical care avoidance behavior (chronic diseases) [5]. Scientists argue that avoiding medical care increases the overall medical expenditure of society and reduces the efficiency of medical resource utilization [6–8]. Underuse of medical care can hinder effective chronic disease management and timely diagnoses, resulting in adverse effects on health [9–11]. Inappropriately high utilization of medical services typically implies an inefficient distribution of healthcare resources and increased healthcare costs, and may be harmful to individuals’ health [12, 13]. Consequently, knowledge of specific psychosocial determinants can inform targeted interventions that aim to manage healthcare use [14]. Therefore, it is important to improve people’s desire to positively use medical care and build their confidence to access services [7, 8].

Studies have shown that owning medical care insurance is an important factor that influences people’s behavioral intention regarding medical care avoidance [3, 9, 15]. Avoidance of medical care emanates from concerns about how far medical care insurance cover regulated costs and how much an individual would need to pay after the state pays, as self-payment of medical care costs is reduced when the insurance cover is generous [16, 17]. However, in most countries, China for example, people are covered either by a public medical care insurance system or a socialized healthcare system, or both; in such cases, provision of medical care insurance is an integral part of the social welfare system [18, 19]. The literature argues that medical care avoidance intention varies with gender differences [20–22]. Men with good health literacy tend to have stronger intention on medical care avoidance than women [23]. However, scientists have found that even when people with low health literacy and are covered by generous social medical insurance, they still have a high possibility of avoiding medical care due to feelings of powerlessness because of dissatisfaction with the medical care policy [22, 24, 25]. People perceive that their expectations from the medical care policy do not match the policy utilization performance. Extant research argues that avoidance of medical care is caused by people’s perceptions and confusion regarding the content of the medical care policy documents, lack of professional knowledge to understand the content, or doubts over medical care policy executors applying the policy effectively due to reasons that they cannot describe [26, 27]. In other words, psychological disconnection from the medical care policy might lead to medical care avoidance [28–30]. Nevertheless, research on how far this psychological disconnection affects medical care avoidance intention is sparse. Therefore, it is necessary to analyze how far medical care avoidance is affected by people’s subjective alienation from medical care policy system, and how far such disconnection from medical care policy and utilization of medical care resources can be addressed, which would have important implications. Thus, this study examines whether this psychological disconnection toward medical care policy—expressed as a sense of policy alienation toward medical care policy (SPA-M)—affects behavioral intention of medical care avoidance (BIA), and to what extent an intermediary variable, medical financial risk perception (MFRP), mediates the relationship between SPA-M and BIA, particularly in countries with a generous medical care policy. This study proposes relevant policy suggestions to reduce medical care avoidance from the perspective of medical care policy alienation.

### Value and significance of this study

This study makes a significant contribution to the literature because it builds on and extends policy alienation literature by focusing on alienation and its effect on medical care policy formulation and implementation. With the ever-increasing cost of providing socialized healthcare insurance, the ramifications of inefficient implementation and underutilization are significant. This study expands the perspective of medical avoidance research and increases the breadth of relevant research since it integrates policy perception and individual medical avoidance tendencies into analysis. It further explores the mechanism between medical care policy alienation and medical care avoidance tendency of middle-aged residents, that is, how policy alienation affects medical avoidance tendency through MFRP.

Given the distinct interpretations of motivational alienation to access medical care resources, the model we construct for this study provides a meaningful parameterization of the relationship between medical care policy alienation and medical care avoidance tendency of middle-aged residents. For the populations for which the model can account for BIA, it not only identifies which factors influence their behavior, but also offers an understanding of *how far* such factors contribute to the reasons of their BIA. With regard to gender differences, this study contributes to enhancing the understanding of the gender issue from a medical avoidance research perspective, providing a basis for the formulation of gender-related interventions.

### Hypotheses development

#### Hypothesis 1

Based on policy alienation research [30–32], this study describes SPA-M as how medical care resources are crafted into people’s cognitive state and the conscious efforts they exert to create experiences using the medical care policy through (a) cognitively recognizing how they view it, and (b) how far they doubt its usefulness. SPA-M should be viewed as a multidimensional construct that aligns the medical care policy with the values and personal medical care needs of people who utilize the policy; it includes the dimensions of meaninglessness, powerlessness, incomprehensibility, implementation doubt, red tape, and technological gap [30, 33]. Based on previous research, we assume that individuals with strong SPA-M may not fully realize the value of medical policies to themselves or their family members (from the perspective of meaninglessness), while they may believe that such a policy cannot be implemented properly by local government (from the perspective of doubt) and that the use of this is so complex that they cannot adapt themselves to it due to empirical reasons (from the perspective of incomprehensibility).

**Fig. 1:**
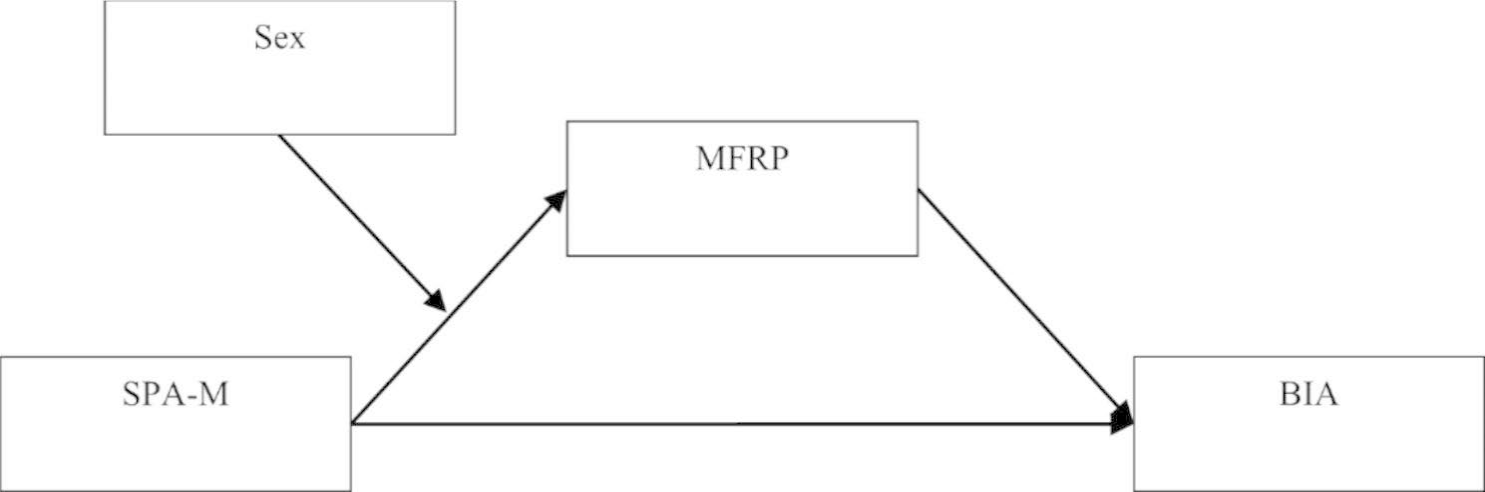
Proposed theoretical model. Legend: SPA-M - Sense of policy alienation toward medical care policy; MFRP - Medical financial risk perception; BIA - Behavior intention of medical care avoidance

Therefore, we propose that SPA-M contributes to the effectiveness of medical care policy utilization, which means that people with strong SPA-M are highly likely to be unwilling to use medical care resources. Therefore, SPA-M is associated with stimulating people’s BIA, which gives us the first hypotheses regarding the link between SPA-M and BIA (see Fig. 1).

##### Hypothesis 1

Individuals with stronger SPA-M have higher BIA.

#### Hypothesis 2

People with a strong SPA-M may be skeptical regarding the extent to which frontline workers (e.g., doctors) implement medical care policy (such as medical care insurance) as they tend to be suspicious of whether or not doctors are providing them with medical treatment at a reasonable cost, or whether all the medical treatment they receive are necessary [34–36]. This makes it difficult for them to obtain comprehensive and sufficient information during their treatment, leading to a higher perception of uncertainty about medical activities that in turn increases their MFRP.

MFRP has been regarded as an important risk factor in various medical risk analysis models [37, 38]. Individuals with high MFRP levels might feel that the perceived benefits of medical treatment are relatively small while the financial costs are high, and that getting medical treatment could affect their normal work–life balance [39–41]. Higher levels of MFRP make it more likely that people will develop BIA. In addition, the literature shows that many people are hesitant to act when they are faced with potentially unpleasant factors in their medical decision-making process and tend to maintain status quo, especially if they have high MFRP [42, 43]. We assume that people’s MFRP could be a mediating variable in the relationship between SPA-M and BIA (see Fig. 1), leading us to the second hypothesis.

##### Hypothesis 2

MFRP plays a mediating role in the relationship between SPA-M and BIA.

#### Hypotheses 3 and 4

Although MFRP plays a mediating role in the relationship between SPA-M and BIA, its impact must also be navigated in terms of sex [21, 22]. According to the literature, males are more sensitive to financial risk perception because they are more perceptive to family and social responsibility [44–46]. If men who need medical care believe that medical care policy is of good value and not too many difficulties are associated with using these policies (i.e., when they have low SPA-M), then they tend to have lower MFRP. In seeking medical help, women are usually relatively likely to engage in help-seeking behaviors than men because such behaviors are less threatening to women’s self-esteem and sex identity than to men’s [22, 29]. In other words, men will experience higher levels of MFRP than women in cases where they both experience insufficient support from medical care policies (i.e., when they have strong SPA-M). Furthermore, compared with women, men are more likely to lack communication skills with doctors and medical workers when they use medical resources [47, 48], which could lead them to experience a sense of uncertainty from getting what they think is less information, which might increase their MFRP [49]. Women have been found to more frequently use medical care resources than men and are more familiar with the medical care system [16, 50]. Even when women have strong SPA-M, they are still able to use care resources smoothly, even if sometimes with a feeling of dissatisfaction [50]. By contrast, men use medical care resources less frequently and their MFRP is more easily influenced by their level of SPA-M. Based on the above analysis, we propose the third and fourth hypotheses (see Fig. 1).

##### Hypothesis 3

Sex moderates the relationship between SPA-M and MFRP; this relationship is stronger for men and weaker for women.

##### Hypothesis 4

Sex moderates the effect of SPA-M on medical care avoidance through MFRP, and this mediating effect is stronger in men.

## Methods

### Sample and procedure

This study aims to examine whether a psychological disconnection toward medical care policy (SPA-M) affects BIA, and to what extent an intermediary variable, MFRP, mediates the relationship between SPA-M and BIA. A cross-sectional study was conducted based on a survey involving 434 middle-aged people (35–59 years) from Wuhu, a city in China’s Anhui province with a medium socioeconomic development level. We selected middle-aged residents aged 35–59 years for analysis because they have increasing medical care needs and high family care responsibility while most of them are also family caregivers [51]. This means that their medical care policy alienation might have great impact on both their own and their family members’ behavior.

We chose Wuhu city as the study focus because its economic and social development is at an average level in China, and it has a high proportion of residency rate in urban area [52]. From a geographical point of view, Wuhu is located on the dividing line between the south and north of China, making it geographically representative. A moderated mediation model was constructed to investigate the research question and sex (biological: male and female) was used as a moderating variable between SPA-M and MFRP based on bootstrapping techniques with M-plus 8.0. Multi-stage sampling method was used to select the respondents. We randomly selected one region from five regions and two counties in Wuhu; we then randomly selected 150 households in each of three communities to complete the questionnaire. With the support of community workers, university student interviewers who understood the local dialect administered the questionnaires. Well-trained interviewers proficient in conducting surveys among residents in urban and rural communities were recruited. They used the convenience sampling method to select the residents based on (1) 35–59 years of age, (2) owning basic medical insurance in the local area, (3) able to communicate with interviewers, and (4) willing to participate in the interview and answer questions after fully understanding the purpose of the study. Most interviewees completed the paper questionnaire individually; however, some of residents required support from the interviewers, who read them the questions and then completed the questionnaire based on their answers. Written consent was obtained from each participant, who received $3 after completing the questionnaire.

This study was approved by the Ethics Committee of Anhui Normal University (approval number: AHNU-ET2022069). A total of 450 questionnaires were distributed and 445 were returned, 14 questionnaires with incomplete answers were excluded, and 434 valid questionnaires were retained for analysis. There were 232 female (53.46%) and 202 male participants (46.54%). Participants had an average age of 50.06 (SD = 5.97); 9.59% had higher education, 18.71% had high school education, 34.05% had junior high school education, 26.38% had primary school education, and 11.27% had no education. A quarter (25.11%) of the participants had extra commercial medical care insurance, in addition to public or social medical care insurance. In the preceding 12 months, 44.65% of the participants (or their family members) had received medical reimbursement (i.e., they had utilized their healthcare policy during the previous year).

### Measures

#### SPA-M

We measured SPA-M using a medical policy alienation scale developed by the authors [53], comprising six dimensions, namely, meaninglessness, powerlessness, incomprehensibility, implementation doubt, red tape, and technological gap, with a total of 23 items, such as “I think frontline policy executors are confused about how to implement a medical care policy” and “I feel helpless when using the electronic medical information system.” Responses were rated on a five-point Likert-type scale (from 1 = strongly disagree to 5 = strongly agree). The reliability and validity of SPA-M scale was tested based on two independent samples of residents (the total sample size was 1174), with exploratory factor analysis and confirmatory factor analysis, before conducting the current study. After negatively worded items were recoded, the average value of the SPA-M scale was used as an indicator of participants’ sense of alienation from medical care policy; the higher the score, the stronger the participant’s sense of policy alienation. Cronbach’s alpha for the SPA-M scale was 0.90.

#### Medical financial risk perception

Referring to Lyu et al. [54], this study used three items to measure participants’ MFRP in the process of using medical resources; participants were asked to recall their last experience of visiting a medical institution (e.g., a hospital). The items were: “During the diagnosis and treatment process, I worried that the hospital would conduct unnecessary tests”; “During the diagnosis and treatment process, I worried that the doctor would give me unnecessary drugs”; and “I worried that I would have to spend too much money during the diagnosis and treatment process.” One factor was extracted from these three items using exploratory factor analysis, which explained 81.32% of the total variation. The commonness of each item was above 0.7 and the factor loads were above 0.8. The average value of the three items was used as the indicator of MFRP; the higher the score, the stronger the MFRP. Cronbach’s alpha for the three items was 0.88 in this study.

#### Medical care avoidance

Following similar research [55], participants were asked to respond to the following item: “I was intent on terminating treatment during the diagnosis and treatment process.” Responses were rated on a five-point Likert-type scale (from 1 = strongly disagree to 5 = strongly agree). Higher scores indicated a stronger intention to avoid medical treatment.

#### Other variables

In the conceptual model, sex was used as a moderating variable and measured as a dichotomous variable (1 = male, 0 = female). According to the literature, age, educational level, and marital status are related to people’s health-seeking behavior [56]. Therefore, this study measured participants’ age, educational level (0 = without any education, 1 = elementary school, 2 = junior high school, 3 = high school, 4 = university), and marital status (1 = married, 0 = other). To control for the influence of health status [57], a seven-point self-rated Likert-type scale was used [58]; the higher the score, the better the health status.

This study included socioeconomic status (SES) as a control variable since it is closely related to health status and health-seeking behaviors [59–61]. The widely used scale developed by Griskevicius et al. [62] was used to measure participants’ SES. This six-item scale has high reliability and validity [63]. The average value of all the items represents the SES index; higher scores indicate higher SES. Cronbach’s alpha for this scale in this study was 0.84. We also measured whether participants had commercial medical insurance in addition to public or social medical insurance (1 = yes, 0 = no) and whether they or their family members had received medical reimbursement in the preceding 12 months (1 = yes, 0 = no) [23].

## Results

### Confirmatory factor analysis

We used confirmatory factor analysis to test the convergence validity of the SPA-M scale. We constructed three models. Model 1 is a single factor model, which means SPA-M was a unidimensional construct, and all 23 items were observational variables of the one potential factor (SPA-M). This model served as a baseline model for comparison with the other models. Model 2 is a 3-factor model, which means SPA-M was an oblique three-dimension model. The first dimension included the items of meaninglessness and powerlessness. The second dimension included the items of incomprehensibility and implemented doubt. The third dimension included the items of red tape and technological gap. Model 3 is a 6-factor model, which means SPA-M was a six-dimension oblique model, and all the six dimensions were correlated. The results show that the fit index of model 3 has reached the standard recommended by relevant experts [64–66], which is significantly better than the other two models (shown in Table 1). Therefore, the data supported the six dimensions of the SPA-M scale, indicating that the scale had high convergence validity.

**Table 1:** Results from the confirmatory factor analysis of the SPA-M scale

Model	*χ* ^2^	*df*	*χ*^2^/*df*	CFI	TLI	RMSEA	SRMR	Δ*χ*^2^(*df)*
Model 1	3706.17	229	16.18	0.45	0.39	0.19	0.14	–
Model 2	2286.95	226	10.12	0.67	0.63	0.15	0.14	1419.22 (3), *p* < .001
Model 3	687.36	214	3.21	0.93	0.91	0.07	0.06	1599.59 (12), *p* < .001

*Note*. *N* = 434. CFI = Comparative fit index; TLI = Tucker-Lewis Index; RMSEA = Root mean square error of approximation; SRMR = Standardized root mean square residual

### Descriptive statistical analysis

Descriptive analysis outcomes (including mean, standard deviation, and correlation coefficient) of SPA-M, MFRP, BIA, and other control variables are presented in Table 2.

**Table 2:** Means (*M*), standard deviations (*SD*), and correlations for study variables (*N* = 434)

Variable	*M*	*SD*	1	2	3	4	5	6	7	8	9	10
1. SPA-M	2.99	0.59										
2. MFRP	3.61	1.09	0.34^***^									
3. BIA	2.63	1.32	0.17^***^	0.36^***^								
4. Sex	0.47	0.50	− 0.12^*^	− 0.08	− 0.05							
5. Age	50.06	5.97	0.17^***^	0.00	0.07	− 0.10^*^						
6. Edu	1.89	1.13	− 0.18^**^	0.03	− 0.14^**^	0.15^**^	− 0.26^***^					
7. Marr	0.96	0.20	0.06	0.11^*^	0.03	− 0.11^*^	− 0.04	0.02				
8. Health	5.19	1.33	− 0.19^***^	− 0.13^**^	− 0.09	0.06	− 0.11^*^	− 0.05	− 0.02			
9. SES	3.10	1.17	− 0.15^**^	− 0.13^*^	− 0.20^***^	− 0.02	− 0.03	0.44^***^	− 0.03	0.09		
10. CoSu	0.25	0.43	− 0.09	0.01	− 0.18^***^	0.09	− 0.15^**^	0.13^*^	0.07	0.00	0.09	
11. Utilize	0.45	0.50	0.05	− 0.04	− 0.01	0.05	− 0.06	0.04	0.12^*^	− 0.11^*^	0.10^*^	0.14^**^

*Note*: * *p* < .05. ** *p* < .01. *** *p* < .001. SPA-M = Sense of policy alienation toward medical care policy; MFRP = Medical financial risk perception; BIA = Behavior intention of medical care avoidance; Edu = Educational level; Marr = Marital status; Health = Self-rated health status; SES = Socioeconomic status; CoSu = Commercial insurance; Utilize = Healthcare policy utilization in the preceding 12 months

The results showed a statistically significant positive correlation between SPA-M and BIA, suggesting that participants with stronger SPA-M may have a higher level of BIA, and a statistically significant positive correlation between SPA-M and MFRP, suggesting that participants with stronger SPA-M may experience higher levels of MFRP in the process of receiving medical treatment. The correlation between MFRP and BIA was statistically significantly positive in this study.

### Common method bias test

We used Harman’s single-factor test to analyze the data to rule out the influence of common method biases in our results [67–69]. We conducted exploratory factor analysis for relevant items for SPA-M, medical care avoidance, and MFRP. The results showed that this factor only explained 20.19% of the total variance, which was much lower than the recommended standard [70, 71] This suggests that common method bias was not a problem in this study.

### Hypothesis testing

To test the four hypotheses, we first analyzed the direct effect of SPA-M on medical care avoidance, and then checked whether MFRP mediated this effect. We constructed a moderated mediation model [72] to determine whether sex moderated the relationship between SPA-M and MFRP, and the extent to which SPA-M differed in its impact on medical care avoidance between the two sexes. We first mean-centered the continuous variables prior to analysis. We used M-plus 8.0 to estimate the parameters of the model [72]. The number of bootstraps was set to 10,000 times.

#### Hypotheses 1 and 2

We found that the coefficient of the impact of SPA-M on medical care avoidance was statistically significant: *B* = 0.38, *p* < .001, 95% CI [0.17, 0.59] (which does not contain zero). Therefore, H1 (*Individuals with stronger SPA-M have higher BIA*) is supported.

Based on this, we found SPA-M had a statistically significant positive effect on the mediating variable MFRP, which means participants with higher levels of SPA-M experienced higher levels of MFRP (see Table 3).

**Table 3:** Results of the simple mediation model (*N =* 376)

Variables	*B*	*SE*	*p*	Bootstrapped 95% CI	
				*LL*	*UL*
Mediator variable model: MFRP					
SPA-M	0.59	0.11	< 0.001	0.37	0.80
Age	-0.01	0.01	0.221	-0.03	0.01
Edu	0.09	0.06	0.096	-0.02	0.20
Marr	0.36	0.31	0.237	-0.39	0.98
Health	-0.05	0.04	0.227	-0.16	0.04
SES	-0.12	0.05	0.021	-0.26	-0.02
CoSu	0.02	0.13	0.852	-0.30	0.27
Utilize	-0.14	0.11	0.198	-0.44	0.07
Outcome variable model: BIA					
SPA-M	0.01	0.13	0.986	-0.25	0.25
MFRP	0.46	0.06	< 0.001	0.34	0.58
Age	0.01	0.01	0.707	-0.02	0.03
Edu	-0.10	0.06	0.107	-0.23	0.02
Marr	-0.20	0.24	0.397	-0.66	0.27
Health	-0.03	0.05	0.595	-0.14	0.08
SES	-0.07	0.06	0.261	-0.19	0.06
CoSu	-0.45	0.14	0.001	-0.73	-0.18
Utilize	0.15	0.12	0.218	-0.09	0.40
SPA-M→MFRP→BIA	0.27	0.07	< 0.001	0.16	0.42

*Note*: CI = confidence interval; *LL* = lower limit; *UL* = upper limit; SPA-M = Sense of policy alienation toward medical care policy; MFRP = Medical financial risk perception; BIA = Behavior intention of medical care avoidance; Edu = Educational level; Marr = Marital status; Health = Self-rated health status; SES = Socio-economic status; CoSu = Commercial insurance; Utilize = Healthcare policy utilization in the preceding 12 months; SPA-M→MFRP→BIA = SPA-M effect on BIA through MFRP.

The results showed that participants’ MFRP had a statistically significantly positive impact on their BIA. Participants with high MFRP have a greater intention to cease medical treatment. More importantly, the effect of SPA-M on medical care avoidance through MFRP was statistically significant at 95% CI, indicating that MFRP mediates the association between SPA-M and medical care avoidance. However, the direct impact of SPA-M on medical care avoidance was not statistically significant after adding the mediator (MFRP) to the model, indicating that MFRP played the role of a complete mediator between SPA-M and medical care avoidance. Consequently, H2 (*MFRP plays a mediating role in the relationship between SPA-M and medical care avoidance*) is supported: participants’ SPA-M levels influenced their BIA through MFRP.

#### Hypothesis 3

The results showed that SPA-M had a statistically significant positive effect on MFRP (*B* = 0.35, *p* = .022, 95% CI [0.06, 0.66]), after we added sex as a factor and the cross-product term between SPA-M and sex (see Table 4). The cross-product term between SPA-M and sex was statistically significant, *B* = 0.46, *p* = .022, 95% CI [0.06, 0.84]. We argue that sex could amplify the effect of SPA-M on MFRP since the interaction term between SPA-M and sex had a positive effect on MFRP, which means that male participants’ SPA-M level had a greater impact on their MFRP than was the case for female participants (male = 1; female = 0).

**Table 4:** Results of the moderated mediation model (*N =* 376)

Variables	*B*	*SE*	*p*	Bootstrapped 95% CI	
				*LL*	*UL*
Mediator variable model: MFRP					
SPA-M	0.35	0.15	0.022	0.06	0.66
Sex	-0.03	0.11	0.805	-0.24	0.18
SPA-M × Sex	0.46	0.20	0.022	0.06	0.84
Age	-0.01	0.01	0.225	-0.03	0.01
Edu	0.09	0.06	0.128	-0.03	0.20
Marr	0.31	0.29	0.293	-0.23	0.91
Health	-0.05	0.04	0.288	-0.13	0.04
SES	-0.13	0.05	0.015	-0.23	-0.03
CoSu	0.04	0.12	0.766	-0.20	0.28
Utilize	-0.12	0.11	0.279	-0.34	0.10
Outcome variable model: BIA					
SPA-M	0.01	0.13	0.979	-0.26	0.26
MFRP	0.46	0.06	< 0.001	0.33	0.38
Sex	0.01	0.13	0.963	-0.24	0.25
Age	0.00	0.01	0.715	-0.02	0.03
Edu	-0.10	0.07	0.110	-0.23	0.02
Marr	-0.20	0.24	0.403	-0.66	0.28
Health	-0.03	0.05	0.595	-0.14	0.08
SES	-0.07	0.06	0.272	-0.19	0.06
CoSu	-0.45	0.14	0.001	-0.73	-0.18
Utilize	0.15	0.12	0.219	-0.09	0.39

*Note*: CI = confidence interval; *LL* = lower limit; *UL* = upper limit; SPA-M = Sense of policy alienation toward medical care policy; MFRP = Medical financial risk perception; BIA = Behavior intention of medical care avoidance; Edu = Educational level; Marr = Marital status; Health = Self-rated health status; SES = Socio-economic status; CoSu = Commercial insurance; Utilize = Healthcare policy utilization in the preceding 12 months; SPA-M→MFRP→BIA = SPA-M effect on BIA through MFRP.

**Fig. 2:**
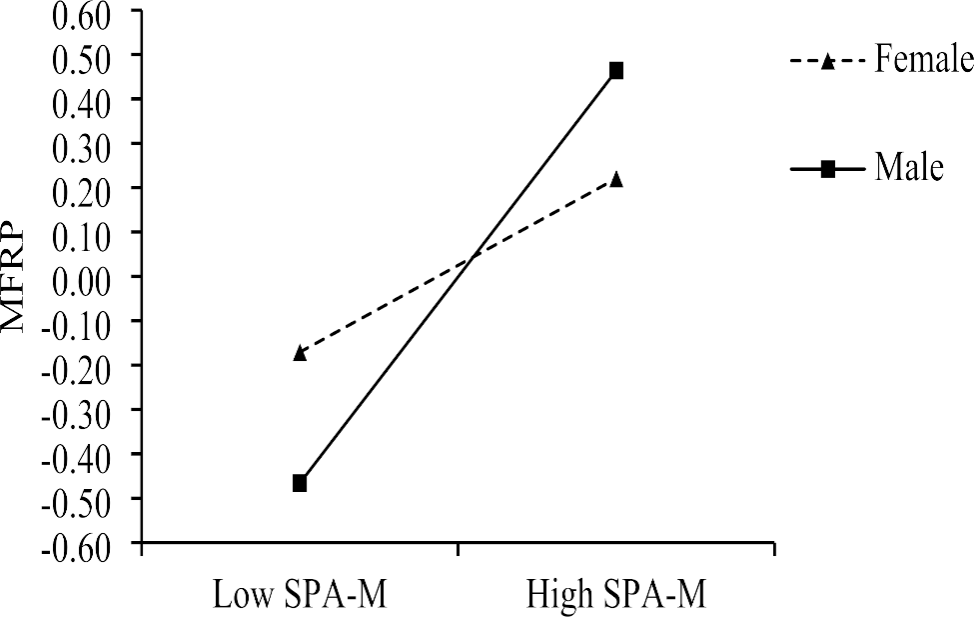
Interaction between SPA-M and sex on MFRP. Sex moderates the relationship between SPA-M and MFRP; the regression coefficient of SPA-M on MFRP is stronger for men and weaker for women. Legend: SPA-M - Sense of policy alienation toward medical care policy; MFRP - Medical financial risk perception

We used simple slope analysis to explore the interaction effect of SPA-M and sex difference in detail [73] (see Fig. 2). For female participants (sex = 0), we found that SPA-M had a statistically significant positive effect on MFRP: *B* = 0.35, *p* = .022, 95% CI [0.06, 0.66]; for male participants (sex = 1), SPA-M had a statistically significant positive effect on MFRP: *B* = 0.81, *p* < .001, 95% CI [0.52, 1.06]. We then constructed an index to represent the differences in the regression coefficients of the relationship between SPA-M and MFRP between male and female participants. The results showed that the 95% confidence interval of the index, calculated by 10,000 bootstraps, was [0.06, 0.84], which does not contain zero, meaning that the regression coefficients of males and females were statistically significantly different. Therefore, H3 (*Sex moderates the relationship between SPA-M and MFRP; this relationship is stronger for men and weaker for women*) is verified, indicating that sex moderated the impact of SPA-M on MFRP.

#### Hypothesis 4

Based on the recommendations of statisticians [74], we examined whether the mediating effect of MFRP between SPA-M and BIA was moderated by sex differences. The results showed that the mediating effect of MFRP on SPA-M and BIA was statistically significantly positive for both female participants: *B* = 0.16, *p* = .035, 95% CI [0.03, 0.33], and male participants: *B* = 0.37, *p* < .001, 95% CI [0.23, 0.54]. We then constructed an index to represent the differences in the mediating effect of MFRP on the relationship between SPA-M and BIA for both sexes. The results showed that the 95% confidence interval of the index, calculated by 10,000 bootstraps, was [0.03, 0.41], which does not include zero. Thus, although the impact of SPA-M on medical care avoidance through MFRP was statistically significant for male and female participants, this mediating effect was greater for male participants. Therefore, H4 (*Sex moderates the effect of SPA-M on medical care avoidance through MFRP, and this mediating effect is stronger in men*) is supported.

#### Outcomes without control variables

Based on the recommendation of scientists [75, 76], we examined whether the mediating effect of SPA-M on BIA through MFRP was still statistically significant without the control variables (including age, health status, SES, and other control variables) in the simple mediation model. The results showed that the mediating effect was still statistically significant without control variables: *B* = 0.26, *p* < .001, 95% CI [0.16, 0.39]. In the moderated mediation model, without control variables, the cross-product term between SPA-M and sex was statistically significant: *B* = 0.47, *p* = .017, 95% CI [0.07, 0.85]). The mediating effect of SPA-M on BIA through MFRP was statistically significant for both women (sex = 0), *B* = 0.16, *p* = .024, 95% CI [0.04, 0.32] and men (sex = 1), *B* = 0.47, *p* < .001, 95% CI [0.23, 0.51]. As for the differences in the mediating effect of SPA-M on BIA through MFRP, when we compared the different sexes, the 95% CI was [0.04, 0.38], which does not contain zero. These results are consistent with the outcomes of our analysis including control variables, indicating robust results [76].

## Discussion

Inappropriate utilization of medical care services typically implies an inefficient distribution of increased healthcare costs with potential adverse effects on health. Knowledge of specific psychosocial determinants can inform targeted interventions that aim to manage medical care use. Improving people’s sense of medical care policy alienation to positively use medical care and build their confidence to access services can stand in the way of effective disease management. Consequently, it is important to analyze whether medical care policy alienation influences people’s medical avoidance tendency since their psychological disconnection from the policy might lead to different BIAs. This study found that SPA-M had a marked impact; the stronger the SPA-M level, the higher the possibility of BIA. We found that MFRP played a fully mediating role between SPA-M and BIA. This means that individuals’ SPA-M could cause them to experience high levels of MFRP, which might result in greater BIA. Furthermore, sex moderated the effect between SPA-M and MFRP; the mediating impact of SPA-M on medical care avoidance was relatively weaker among female participants than among male participants.

### Practical implications

The results provide insights for a better understanding of the impact factors behind people’s behavioral intention toward medical care avoidance. Previous research has focused more on how well people are covered by medical care insurance and how much they have to pay for medical costs for the medical care insurance beyond the state’s share. However, this study highlights that generous medical care insurance does decrease medical care avoidance intention of middle-old aged population owing to SPA-M. It is vital that the government pays more attention to medical care policy alienation to improve people’s medical care policy utilization efficiency. Previous research has shown that psychological disconnection from medical care policy leads to interruptions in receiving continuous medical treatment and damages people’s trust in medical care institutions and doctors, which is contradictory to the main purpose of national healthcare services [31, 77, 78]. While we argue that it is important to understand people’s BIA and its impact factors so that people can use medical care resources uninterruptedly in a way they prefer, it is also important to deliver these resources in more effective ways.

Furthermore, we propose a greater consideration of the role of sex differences in the analysis of the relationship between SPA-M, MFRP, and BIA, particularly for practitioners. Our study enhances the literature on the impact of sex differences on the analysis of the relationship between SPA-M and BIA; such impact indirectly expressed itself through the mediating variable MFRP [22, 79]. We argue that reducing men’s SPA-M level could be an effective way to mitigate their reluctance to engage in medical help-seeking behaviors. It is necessary to implement medical care policies by focusing on sex difference strategies, and link the relationship between medical care policy implementation and medical help-seeking behaviors in different groups of people. For men, medical care avoidance might have great impact on family decisions about using medical care resources, which might lead to changes in the family’s quality of life, more so in rural areas where men are generally the breadwinners and head of the family. Therefore, men’s SPA-M level should be carefully observed in practice in China.

### Theoretical contribution

First, this study contributes to the existing literature on the understanding of the role of SPA-M in medical care policy. We found that the higher people’s SPA-M, the more likely they are to avoid medical care, and the more negative attitudes they have toward medical institutions and workers because of their concerns about the implementation and effectiveness of the medical care policy [8]. Some studies have found lack of social support (such as lack of medical care insurance) to be an important reason people avoid or stop medical treatment [17, 76]. As this study explores the mechanisms by which policy alienation from individual healthcare policies affects medical avoidance, it has important implications for people’s well-being, effectiveness of healthcare policy utilization, and utilization of healthcare resources. Our findings provide further evidence of why feelings of alienation from the policy leads to avoidance or cessation of medical treatment, even with insurance cover (often more than one, such as private insurance). Second, this study contributes to policy alienation theory by presenting medical care policy alienation from the perspective of residents instead of policy executors. It shows that policy alienation can exist even in terms of policy utilization, providing support to the argument that policy alienation needs to be included as a significant factor that influence policy making process, from the perspective of policy users. Third, this study contributes to medical care avoidance theory by revealing that a new mediator can affect people’s potential medical care avoidance behavior intention, and sex also plays an important role in the extent of care avoidance actions.

### Recommendations

First, it is important to re-examine the uneven distribution of medical care resources, which has long been cited as an explanatory factor for policy utilization inefficiency. However, Chinese reforms aimed at this problem have not received good results. Therefore, it is necessary to rationally and scientifically analyze the impact of the allocation of medical care resources on residents’ medical care policy alienation, and systematically analyze the relationship between medical care resources utilization and medical policy implementation according to the characteristics of policy alienation. Instead of the existing “positive thinking,” the government needs to switch to “reverse thinking,” to explore what kind of medical care resources allocation is needed to reduce medical care policy alienation and improve the efficiency of social policy utilization.

Second, it is significant to pay attention to residents’ experience of using medical care policy, such as medical treatment and old age care experience, which can profoundly affect the spillover results of their medical care policy utilization, in turn influencing policy implementation. Residents’ experience sharing and dissemination of medical care policy utilization has a wide-ranging impact on their social group. This helps those who implement the policy at the grassroots level to gain a detailed understanding of the difficulties they encounter in the policy utilization process, and their experiences, feelings, and opinions to not only expand the influence of public opinion of high-quality experience but also help eliminate the negative impact of policy alienation.

Third, it is necessary to examine the influence of traditional social customs. Residents’ sense of acquisition of medical care policy is an important factor affecting their policy alienation. The sense of medical care policy acquisition largely depends on the accessibility of medical care resources. However, traditional customs, as moderating variables in this path, can affect residents’ sense of acquisition of medical care policy through boundary conditions. Therefore, it is recommended to pay attention to the role of traditional customs in alienating residents from medical care policy in different countries, separate respect for customs from policy use behavior, and separate residents’ compliance with traditional customs from the actual transformation of medical care policy resources.

### Limitations

The study has certain limitations. To begin with, its cross-sectional design means that causal inferences cannot be made. Future research could consider using a longitudinal design, such as a cross-lagged design, to explore possible causal relationships between SPA-M and medical care avoidance. Second, this study focused on the outcome variable as behavioral intention toward medical care avoidance, which is a behavioral tendency but is theoretically different from medical care avoidance behavior. Although behavioral intention is one of the key factors affecting behavior, according to psychological theories such as the theory of planned behavior [80, 81], behavioral tendency may not be completely equal to avoidance behavior. Future research should include more objective behavioral indicators and integrate multiple types of data (such as medical records from medical institutions) to explore the impact of medical policy alienation on medical care avoidance behavior. Third, this study does not exclude the impact of other mediating variables besides MFRP. For example, it is possible that individuals’ SPA-M influences their trust in medical institutions, in turn affecting their BIA. Future research should explore in depth the mechanisms of how far SPA-M affects individuals’ medical help-seeking activities. Fourth, this study only focused on participants aged 35–59, and future research should broaden the targeted age group. Finally, more in-depth investigations can be carried out in other regions of China and other countries in the future instead of only focusing on Wuhu.

## Conclusions

This study investigates whether the SPA-M of individuals affects BIA, and to what extent MFRP mediates the relationship between SPA-M and BIA. Given the adverse effects that cessation or avoidance of medical care can have on health status, understanding why people—even those with medical care insurance—choose to avoid or stop treatment is important from the perspective of both the individual and stakeholders, especially those formulating or implementing medical care policy. We found that SPA-M had a marked impact on individuals’ BIA and that this relationship was fully mediated by MFRP, associated with medical costs and moderated by sex (male and female) differences; for men, this relationship is stronger, and for women, it is weaker. The study provides evidence for and insights into the intersection between elements of policy formulation and implementation, and public perceptions of shortcomings and risks involved in public health policy issues.

## Data Availability

The datasets generated and/or analyzed during the current study are not publicly available. The anonymized data can be obtained from two sources. First, access to the anonymized data is available from the local government, which provided financial support for this study. Second, it is available from the corresponding author upon reasonable request and with the permission of the School of Educational Science, Anhui Normal University in China.

## List of abbreviations

SPA-M: Sense of policy alienation toward medical care policy
BIA: Behavior intention of medical care avoidance
MFRP: Medical financial risk perception
SES: Socioeconomic status
